# Involvement of impaired autophagy and mitophagy in Neuro-2a cell damage under hypoxic and/or high-glucose conditions

**DOI:** 10.1038/s41598-018-20162-1

**Published:** 2018-02-19

**Authors:** Yufei Song, Yu Du, Wenying Zou, Yan Luo, Xiaojie Zhang, Jianliang Fu

**Affiliations:** Department of Neurology, Shanghai Jiao Tong University Affiliated Sixth People’s Hospital, 600 Yishan Road, Shanghai, 200233 China

## Abstract

Chronic cerebral hypoperfusion (CCH) plays an insidious role in the development of cognitive impairment. Considerable evidence suggests that Diabetes Mellitus (DM) as a vascular risk factor may exacerbate CCH and is closely related to cognitive decline. Dysregulation of autophagy is known to be associated with the pathogenesis of neurodegenerative diseases such as Alzheimer’s disease. To elucidate the role of autophagy in CCH- and/or DM-related pathogenesis, mouse neuroblastoma Neuro-2a cells were exposed to hypoxia and/or high glucose for 48 h, mimicking CCH complicated with DM pathologies. Chronic hypoxia reduced cell proliferation and increased levels of cleaved caspase-3, whereas high glucose had no obvious synergistic toxic effect. Accumulation of autophagic vacuoles under hypoxia may be due to both autophagy impairment and induction, with the former accounting for Neuro-2a cell death. Additionally, aberrant accumulation of mitochondria in Neuro-2a cells may be attributed to insufficient BNIP3-mediated mitophagy due to poor interaction between BNIP3 and LC3-II. Despite the lack of a significant cytotoxic effect of high glucose under our experimental conditions, our data indicated for the first time that impaired autophagy degradation and inefficient BNIP3-mediated mitophagy may constitute mechanisms underlying neuronal cell damage during chronic hypoxia.

## Introduction

Chronic cerebral hypoperfusion (CCH) is a normal process related to ageing that likely contributes to age-related memory loss^1^. Nevertheless, multiple vascular risk factors, such as hypertension, Diabetes Mellitus (DM), atherosclerosis and hypercholesterolemia, will accelerate the rate of cerebral blood flow decline to a consequential threshold, leading to an insidious conversion of age-related forgetfulness to dementia, a pathological pathway that emerges in both Alzheimer’s disease (AD) and vascular dementia (VaD)^2,3^. A chronic reduction in cerebral blood supply induces neuroinflammation, oxidative stress, white matter lesions, and hippocampal and neuronal degeneration/death, all of which lead to cognitive dysfunction^4^. DM, one of the most common vascular factors, has been reported to be closely associated with cognitive impairment^5^; moreover, its characteristic event, hyperglycaemia, with an increase in neuronal glucose levels of up to fourfold, has been reported to gradually induce neuronal dysregulation and structural abnormalities in the brain^6^. However, whether hyperglycaemia exacerbates the pathologies of CCH remains unclear, as do the underlying mechanisms through which this occurs. In contrast to the considerable evidence for the cellular mechanisms by which acute ischaemia affects the brain^7,8^, less is known about the consequences of CCH and/or DM towards it.

Autophagy is a digestion pathway through which bulk degradation of cytosolic components and organelles occurs; the process includes double-membrane autophagosome formation, fusion with a lysosome, and ultimately degradation of cargo by lysosomal enzymes. Microtubule-associated protein1 light chain 3 (LC3-I) plays critical roles in both autophagosome membrane formation and target recognition. LC3-I is converted to a phosphatidyl ethanolamine (PE)-conjugated LC3-II form in the initial autophagy process of phagophore biogenesis. The polyubiquitin-binding protein P62, which tags misfolded proteins and unwanted organelles, is selectively recruited to phagophores. P62 directly binds to LC3 through the specific LC3-interaction region (LIR), leading to its efficient degradation via autophagy^9^. Dysregulation of autophagy has been linked to the pathogenesis of neurodegenerative diseases such as AD, which is characterized by progressive cognitive decline. Defects in the transport and/or acidification of autophagic vacuoles (AVs) block the removal of amyloid-β (Aβ) by lysosomes, in turn exacerbating Aβ deposition^10^. Moreover, hypoxia has long been known to trigger autophagy in both *in vivo* and *in vitro* models of acute or transient ischaemic brain injury^11,12^. AMP-activated protein kinase (AMPK), an intracellular sensor of ATP storage, is activated during hypoxia and starvation which can inhibit a central suppressor of autophagy, rapamycin complex 1 (mTORC1), and result in enhanced upregulation of autophagy^13^. Many studies have reported a neuroprotective role for autophagy in acute brain ischaemia^14^. However, Its role in the pathologies of CCH-related cognitive impairment remains unclear.

As neurons require a high energy supply, mitochondria which are the main resource of cellular energy via oxidative phosphorylation play a vital role in neuronal function. Nonetheless, the toxic byproducts of oxidative phosphorylation including reactive oxygen species (ROS) also induce oxidative damage to mitochondria, in turn triggering the organelles to produce more ROS and leading to a release of cytochrome c and cellular injury^15^. Notably, mitochondrial damage has been implicated in neurodegenerative diseases, including AD and Parkinson’s disease (PD)^16^. Indeed, a clearance of damaged mitochondria and a guaranteed number of intact mitochondria are imperative to cellular viability. The elimination of old and damaged mitochondria mainly occurs through mitophagy, a selective form of autophagy, and this process is responsible for not only basal mitochondrial quality control but also a stress-response mechanism, such as in response to hypoxic conditions, to selectively eliminate injured mitochondria^17^. Researchers have described two basic pathways of mitophagy: receptor-dependent and receptor-independent mechanisms. The former includes BCL-2/adenovirus E1B (19 K)-interacting protein (BNIP3)-mediated mitophagy. BNIP3, which is upregulated via hypoxia-inducible factor-1 (HIF-1), was initially described as a mitochondrial outer membrane protein that promotes programmed cell death by inducing cytochrome c release. Later, BNIP3 was also shown could eliminate damaged mitochondria as a mitophagy receptor via interaction with LC3-II, and numerous studies have shown that BNIP3 can either play a protective or detrimental role, depending on the cell type and cellular environment. The receptor-independent mitophagy pathway is well known as PTEN-induced putative kinase 1 (PINK1)/Parkin-mediated mitophagy, which participates in the response to eliminate depolarized mitochondria. PINK1 is imported from the cytosol to mitochondria via its mitochondrial targeting sequence where it is cleaved by mitochondrial matrix proteases under basal conditions. Upon loss of mitochondrial membrane potential (Δψm), PINK1 is stabilized instead at the mitochondrial outer membrane to recruit Parkin into target mitochondria, promoting the removal of damaged mitochondrial via mitophagy. Inefficient mitophagy induces accumulation of dysfunctional mitochondria, contributing to the pathogenesis of diseases characterized by dysfunctional energy metabolism^18,19^.

To illustrate whether hyperglycaemia exacerbates the pathologies of CCH and whether autophagy and mitophagy mechanisms are involved in the process, mouse neuroblastoma Neuro-2a cells were exposed to hypoxia and/or high glucose to mimic the pathologies of CCH complicated with DM. The results of our study showed that chronic hypoxia does in fact induce Neuro-2a cell death, though high glucose did not have an obvious synergistic toxic effect. We also found AV accumulation with hypoxia, perhaps due to both impaired autophagic degradation and increased autophagic induction, with the former being a reason for the reduced viability of Neuro-2a cells. In addition, inefficient BNIP-mediated mitophagy may contribute to the accumulation of aberrant mitochondria that is observed in Neuro-2a cells during chronic hypoxia.

## Results

### Chronic hypoxia induced Neuro-2a cell death, whereas high glucose did not have a marked toxic effect

**Figure 1:**
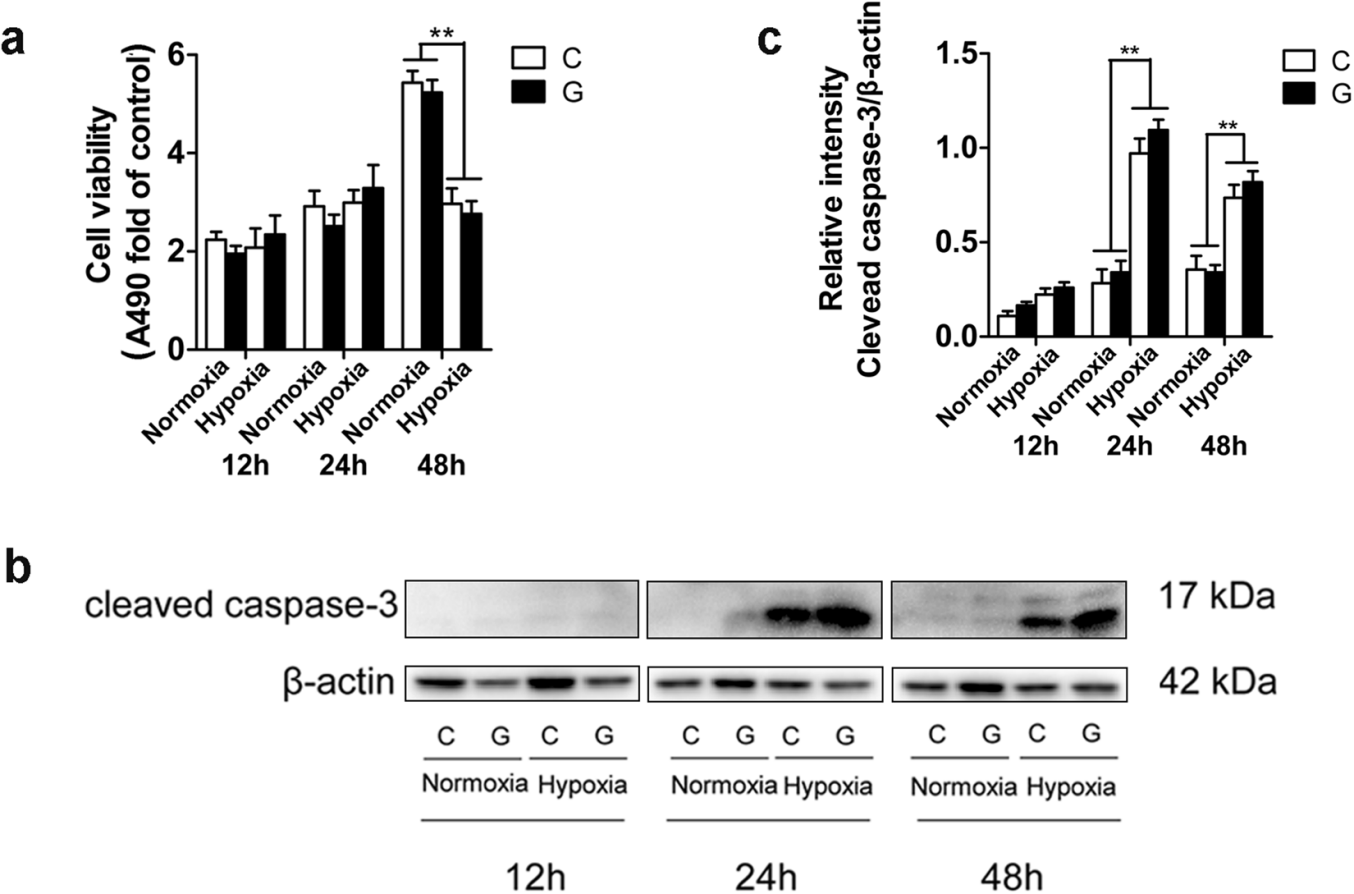
Effect of chronic hypoxia and/or high glucose on Neuro-2a cell viability. (**a**) Measurement of Neuro-2a cell proliferation. Neuro-2a cells were exposed to hypoxia and/or high glucose for up to 48 h. “C” represented control conditions (25 mM glucose), “G” represented high glucose conditions (75 mM glucose). Cell proliferation was determined by the MTS assay. (**b**,**c**) Western blot analysis of cleaved caspase-3 expression in Neuro-2a cells. The ImageJ densitometric analysis of the cleaved caspase-3 from immunoblots is shown. The results were expressed as the mean ± SEM from three independent experiments. Two-way ANOVA, **p < 0.01, Hypoxia vs Normoxia. Full-length blots are presented in Supplementary Fig. S2.

To investigate whether chronic hypoxia and/or high glucose can reduce cell viability, Neuro-2a cells were exposed to hypoxia and high glucose separately or together for up to 48 h. We found that 24 h of hypoxia treatment led to cell detachment, vacuolization and shrinkage and notably decreased the number of cells at 48 h. In contrast, high glucose failed to obviously alter cell morphology (Supplementary Fig. S1). Similarly, a 3-(4,5-dimethylthiazol-2-yl)-5-(3-carboxymethoxyphenyl)-2-(4-sulfophenyl)-2H-tetrazolium (MTS) assay revealed that exposure to hypoxia markedly reduced Neuro-2a cell proliferation after 48 h. High glucose was not markedly toxic towards Neuro-2a cells, even though it exerted a potential detrimental effect on cell proliferation during hypoxia, as demonstrated by a marginal but not significant reduction in optical density (OD) (Fig. 1a). To further characterize this hypoxia-induced cell injury, we employed immunoblotting to assess the level of the apoptosis-related protein cleaved caspase-3 under hypoxic and/or high-glucose conditions. As shown in Fig. 1b,c, the level of cleaved caspase-3 increased dramatically in Neuro-2a cells during hypoxia at 24 h and 48 h, though the protein level was slightly lower at 48 h than at 24 h, perhaps due to protein degradation during the longer duration. Just like the results form Fig. 1a, there was also a slight but not dramatic increase in cleaved caspase-3 levels with hypoxia plus high-glucose treatment compared with that observed with hypoxia alone. These results demonstrate that chronic hypoxia markedly induced Neuro-2a cell injury, whereas high glucose did not result in a significant cytotoxic effect on cells with or without hypoxia.

**Figure 2:**
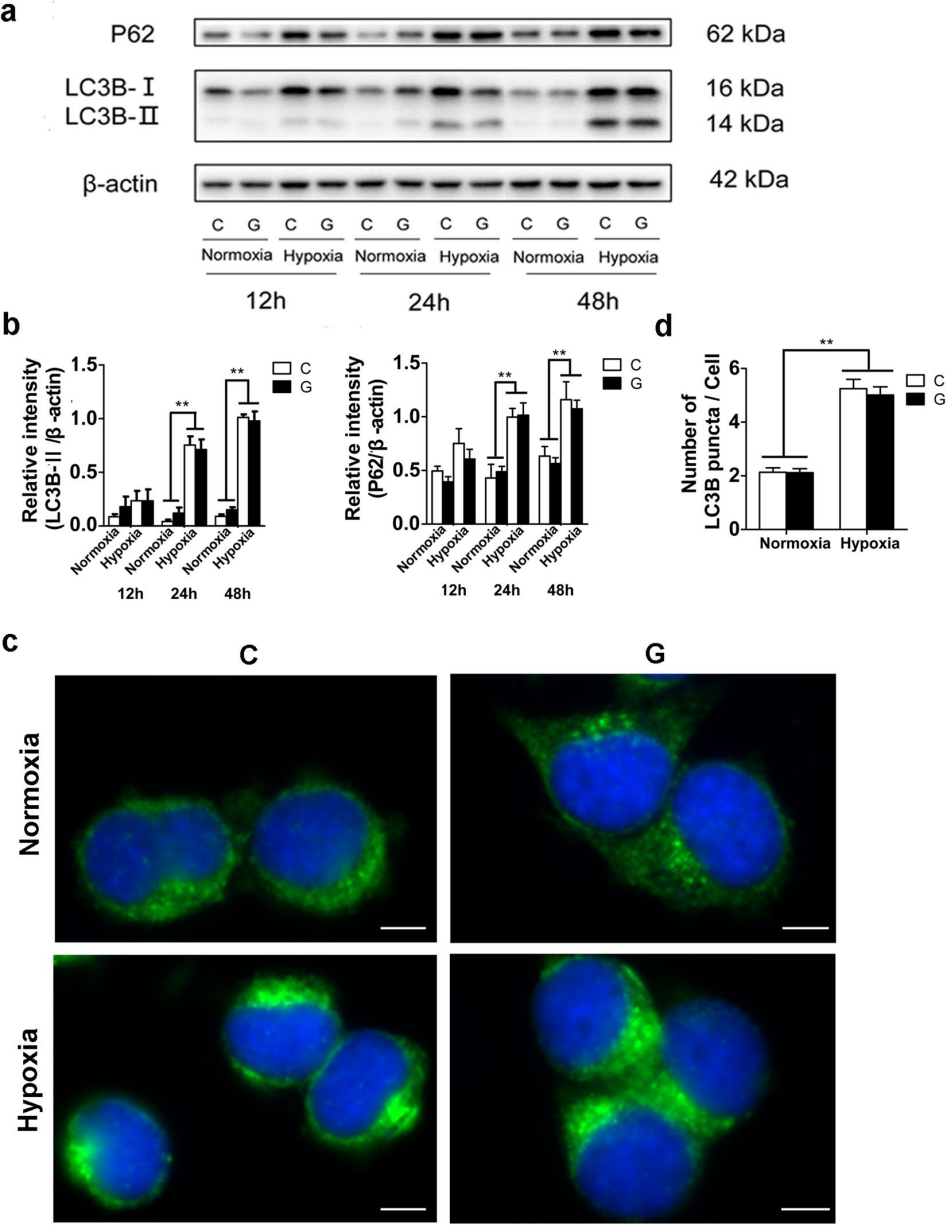
Accumulation of AVs under hypoxic stress. (**a**) Neuro-2a cells were submitted to hypoxia and/or high glucose for 48 h, and the protein levels of P62 and LC3B-II were measured by immunoblots, showing a time-dependent increase in levels of LC3-II and P62 under hypoxia compared with those under normoxia. In contrast, high glucose had no significant effect on their expression. (**b**) The ImageJ densitometric analysis of P62 and LC3B-II from the immunoblots is shown. “C” represented control conditions (25 mM glucose), “G” represented high glucose conditions (75 mM glucose). The results are expressed as the mean ± SEM from three independent experiments. Two-way ANOVA, **p < 0.01, Hypoxia vs Normoxia. (**c**) Immunofluorescent assay for the endogenous distribution and localization of LC3B in Neuro-2a cells. Neuro-2a cells were submitted to hypoxia and/or high glucose for 48 h and then fixed. LC3B was visualized as a green signal, and the blue sections are nuclei stained by DAPI. Scale bar, 20 μm. (**d**) Histogram shows the number of AVs per cell (LC3B-positive puncta) in Neuro-2a cells. The results are expressed as the mean ± SEM from three independent experiments with nine microscopy fields for each experiment. Two-way ANOVA, **p < 0.01, Hypoxia vs Normoxia. Full-length blots are presented in Supplementary Fig. S2.

### Chronic hypoxia contributed to accumulation of AVs

**Figure 3:**
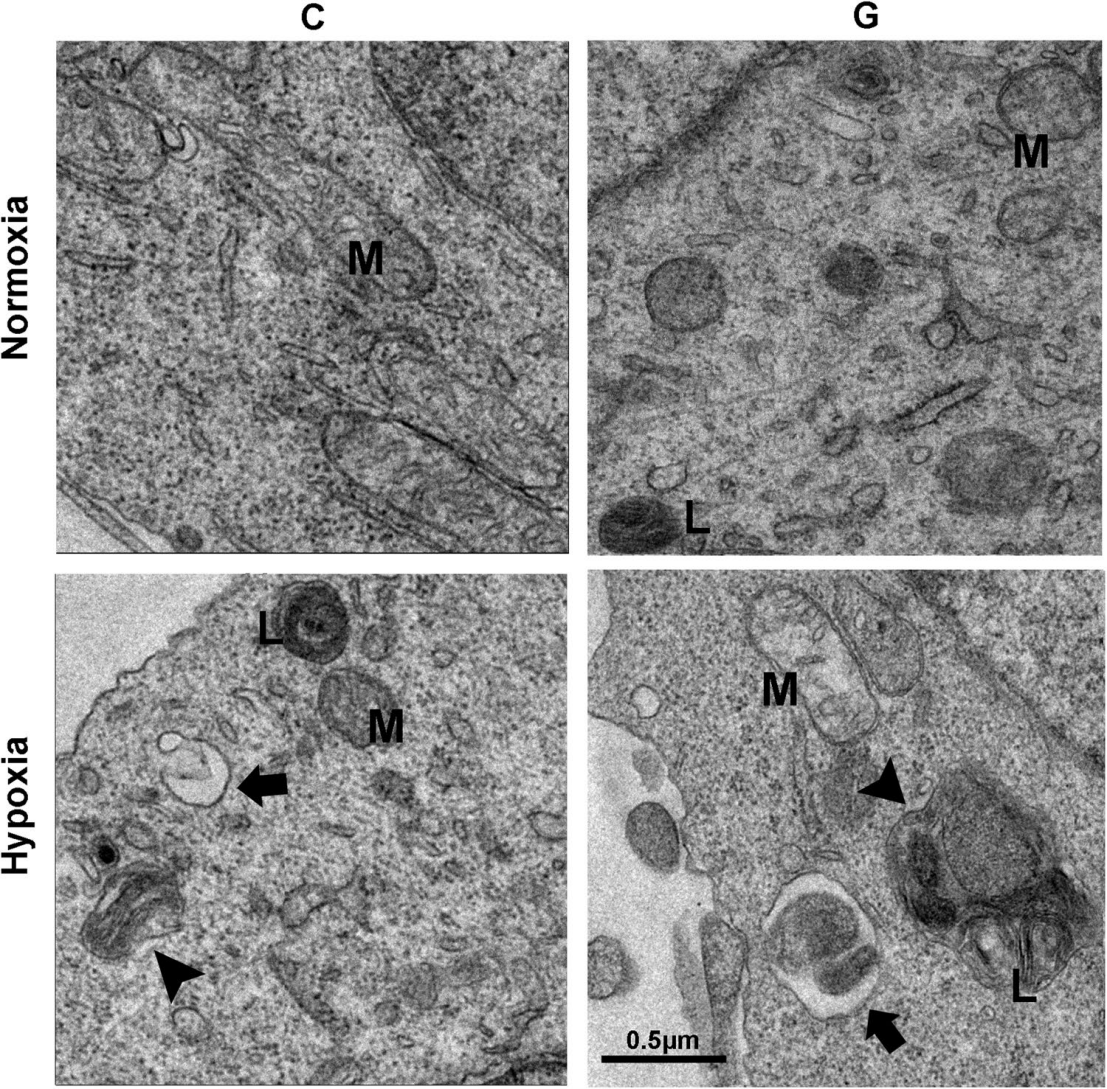
Electron microscopy showing the increased number of AVs. “C” represented control conditions (25 mM glucose), “G” represented high glucose conditions (75 mM glucose). Arrowheads indicate the initial autophagic vacuoles; Arrows represent the degrading autophagic vacuoles. M indicate mitochondria; L represent lipid droplets. Scale bar: 0.5 μm.

LC3 is required for the formation of autophagosome membranes and is modified from LC3-I to LC3-II during autophagy activation, the latter of which is considered a marker of autophagosomes in mammalian cells. Sequestosome-1 (SQSTM1)/P62 is a poly-ubiquitin-binding protein that is degraded via autophagy; therefore, the protein level of P62 is inversely related to autophagic activity. These two markers are used to evaluate the effect of hypoxia and/or high glucose on autophagy in Neuro-2a cells. As demonstrated by the immunoblot analysis shown in Fig. 2a,b, we found a time-dependent increase in the levels of LC3-II and P62 under hypoxia compared with those under normoxic conditions. However, high glucose had no significant effect on autophagy, as demonstrated by a slight decrease in the level of LC3-II under the hypoxic and high-glucose condition compared with that in cells treated with hypoxia alone. Moreover, hypoxia-treated Neuro-2a cells exhibited a greater accumulation of AVs than under the normoxic conditions, as revealed by an increase in the number of LC3-positive dots per cell according to immunocytochemistry analysis (Fig. 2c,d). AVs include double-membrane autophagosomes and single-membrane autolysosomes; the inner membrane of the former disappears after fusion with a lysosome, which is then considered an autolysosome. As expected, transmission electron microscopy (TEM) revealed more AVs in Neuro-2a cells under hypoxic than normoxic conditions (Fig. 3). Taken together, these results clearly demonstrate that chronic hypoxia treatment induced an increase in AVs in Neuro-2a cells.

### Accumulation of AVs under hypoxia was due to both autophagy impairment and autophagy induction

**Figure 4:**
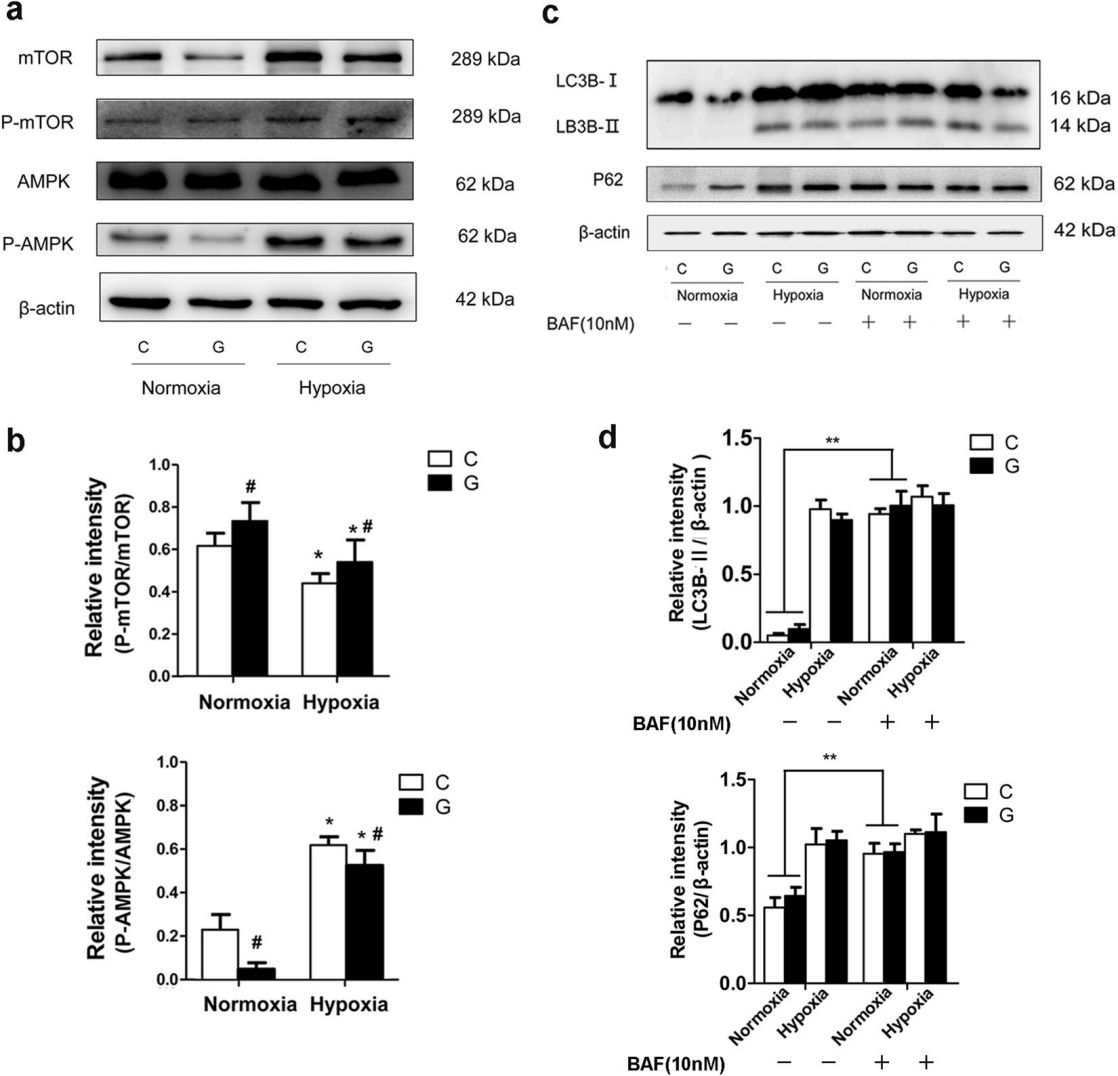
Autophagy impairment and autophagy induction under chronic hypoxia. (**a**) Neuro-2a cells were exposed to hypoxia and/or high glucose for 48 h, and the protein levels of P-AMPK, AMPK, P-mTOR and mTOR were measured by immunoblot. (**b**) The ImageJ densitometric analysis showed that chronic hypoxia upregulated the ratio of P-AMPK/AMPK and lowered the ratio of P-mTOR/ mTOR when compared with those under normoxia. High glucose decreased the ratio of P-AMPK/AMPK. “C” represented control conditions (25 mM glucose), “G” represented high glucose conditions (75 mM glucose). The results are expressed as the mean ± SEM from three independent experiments. Two-way ANOVA, ^#^*P* < 0.05 High Glucose vs. Control; *P < 0.05 Hypoxia vs. Normoxia. (**c**) Neuro-2a cells were submitted to hypoxia and/or high glucose for up to 42 h and then subjected to 10 nM BAF for an additional 6 h. Immunoblots were performed to determine the levels of P62 and LC3B-II proteins in Neuro-2a cells. (**d**) The ImageJ densitometric analysis showed that BAF treatment increased the protein levels of LC3-II and P62 in Neuro-2a cells under normoxia but did not further change the expression of LC3-II and P62 under hypoxia. The results are expressed as the mean ± SEM from three independent experiments. Two-way ANOVA, **p < 0.01 treated vs. untreated. Full-length blots are presented in Supplementary Fig. S2.

The increased levels of LC3-II and P62 proteins under hypoxia indicated that the observed higher number of AVs maybe the result of impaired autophagic degradation and/or increased autophagic induction. To address this, we first examined whether autophagy is promoted under hypoxic and/or high-glucose conditions. Through inhibition of mTORC1, the intracellular energy sensor AMPK serves as an indirect positive regulator of autophagy; unphosphorylated AMPK is inactivated, whereas the phosphorylation at Thr172 activates it under conditions of ATP shortage. Thus, we assessed the protein levels of phosphorylated AMPK (P-AMPK), AMPK, P-mTOR and mTOR via immunoblotting of Neuro-2a cells exposed to hypoxia and/or high glucose for 48 h. As expected, hypoxia, which led to a lack of energy, induced autophagy, as illustrated by a significantly higher ratio of P-AMPK/AMPK and a lower ratio of P-mTOR/ mTOR than under the normoxic conditions (Fig. 4a,b). Moreover, high glucose decreased the level of phosphorylated AMPK, which is also a possible explanation for the lower level of LC3-II in cells treated with hypoxia and high glucose than in those treated with hypoxia alone (Fig. 2b).

Next, we investigated whether impairment in autophagy flux was involved. Bafilomycin A1 (BAF) is a selective inhibitor of H+-ATPase, which inhibits the degradation of autophagosomes^20^. Neuro-2a cells were subjected to hypoxia and/or high glucose for up to 42 h and then treated with 10 nM BAF for an additional 6 h. Notably, western blot analysis clearly showed that the protein levels of LC3-II and P62 increased in these cells under normoxic conditions in the presence of BAF. However, the hypoxia-induced accumulation of LC3-II and P62 was not significantly enhanced by BAF, indicating that chronic hypoxia blocked autophagy prior to BAF treatment (Fig. 4c,d).

Collectively, these observations indicate that hypoxia-induced AV accumulation maybe due to both impaired autophagic degradation and increased autophagic induction.

### Impaired autophagy during chronic hypoxia caused Neuro-2a cell death

**Figure 5:**
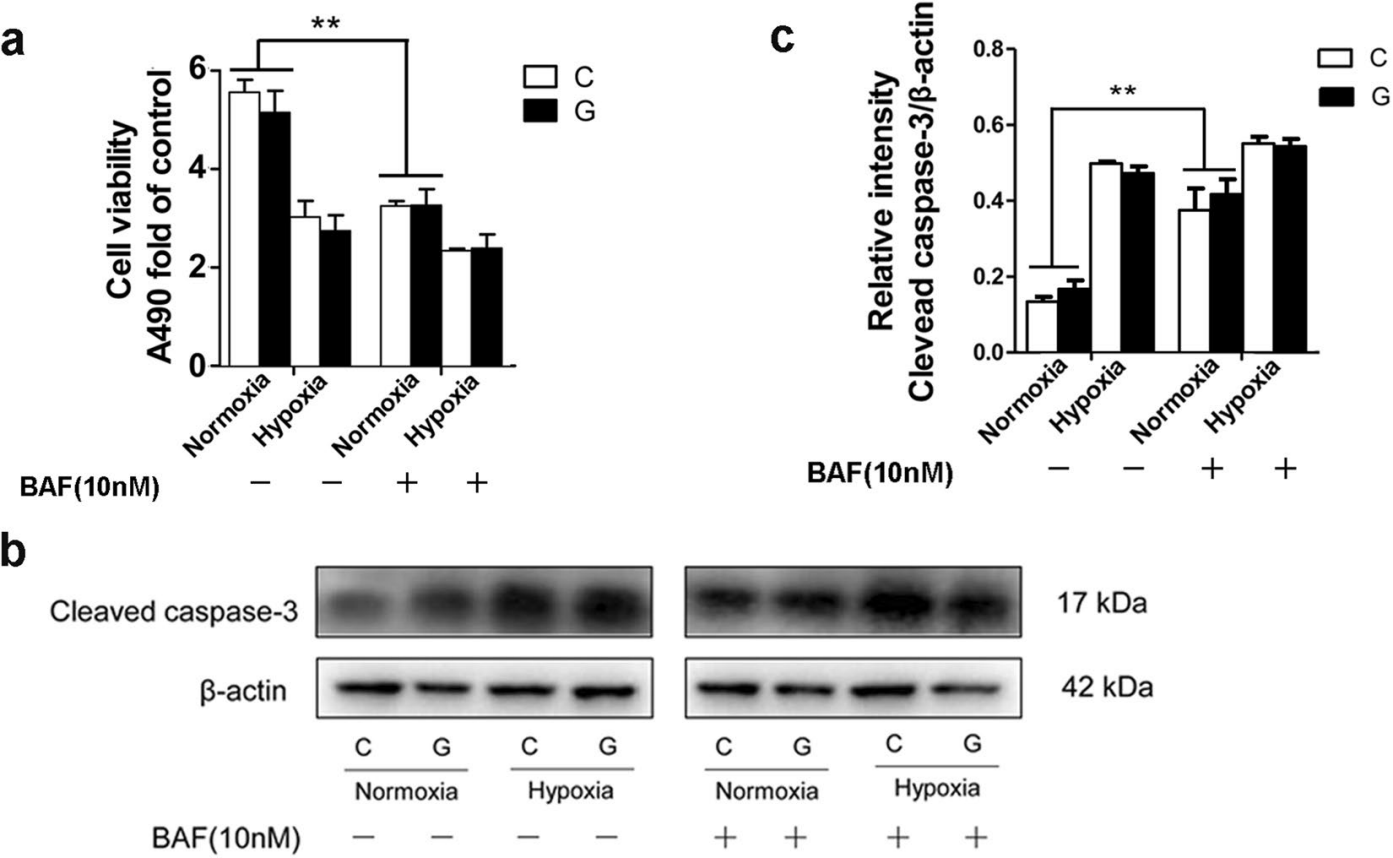
Inhibition of autophagy degradation induced cell death. (**a**) An MTS assay was used to access Neuro-2a cell proliferation under hypoxia and/or normoxia in the presence or absence of BAF. Treatment with BAF induced a decreased OD value under normoxia but did not further change the OD value under hypoxia. “C” represented control conditions (25 mM glucose), “G” represented high glucose conditions (75 mM glucose). (**b**) Immunoblots were performed to determine the level of the cleaved caspase-3 protein in Neuro-2a cells. BAF treatment induced an increase in the level of cleaved caspase-3 protein under normoxia but did not further change the expression of cleaved caspase-3 under hypoxia. The results are expressed as the mean ± SEM from three independent experiments. Two-way ANOVA, **p < 0.01 treated vs. untreated. Full-length blots are presented in Supplementary Fig. S2.

As our data suggested an autophagy impairment in Neuro-2a cells exposed to hypoxia, we then evaluated the effect of autophagy blockade on Neuro-2a cell death. BAF treatment had a significant cytotoxic effect, as demonstrated by a significantly lower OD value in the MTS assay and higher expression of cleaved caspase-3 than in untreated cells under normoxic conditions, whereas BAF treatment did not further limit cell survival under hypoxia (Fig. 5). Together, these results indicate that impaired autophagy flux likely contributed to AV accumulation and Neuro-2a cell death during chronic hypoxia.

### Chronic hypoxia injured mitochondria in Neuro-2a cells

**Figure 6:**
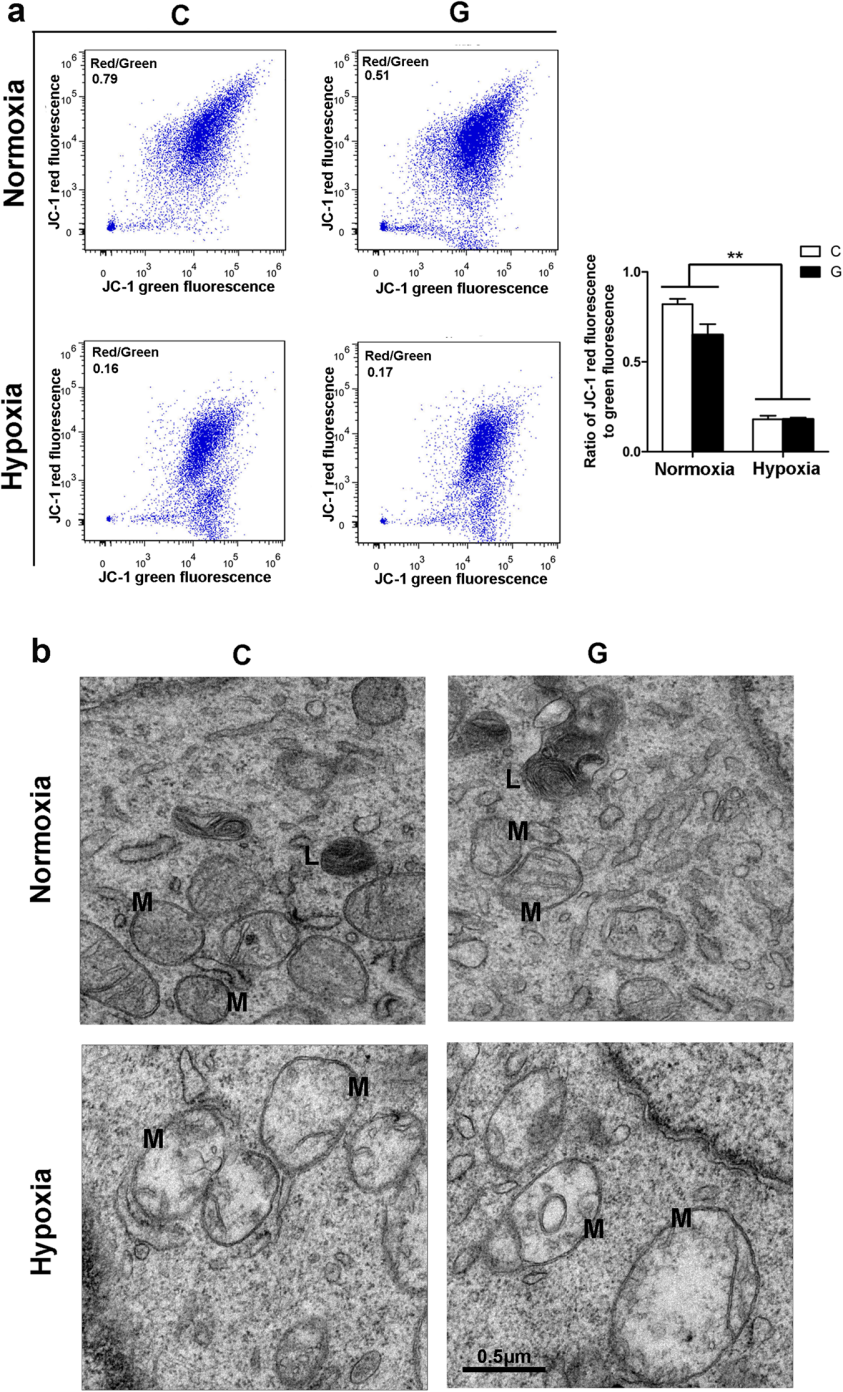
Chronic hypoxia triggered a loss of mitochondrial potential and destruction of the mitochondrial structure. (**a**) Neuro-2a cells were subjected to hypoxia and/or high glucose for up to 48 h. The mitochondria potential was measured by JC1 staining. Quantitative analysis by flow cytometry revealed that chronic hypoxia lowered the ratio of the red and green fluorescence, while high glucose did not change it with or without hypoxia. “C” represented control conditions (25 mM glucose), “G” represented high glucose conditions (75 mM glucose). The results are expressed as the mean ± SEM from three independent experiments. Two-way ANOVA, **p < 0.01, Hypoxia vs Normoxia. (**b**) Representative examples of mitochondrial morphology under hypoxia and/or high glucose examined via TEM. M indicate mitochondria; L represent lipid droplets. Scale bar: 0.5 μm.

Mitochondria are cellular powerhouses that produce ATP by oxidative phosphorylation. As neurons require large amounts of energy, it is not surprising that mitochondria homeostasis is strictly essential for an optimally functioning neurons. Accordingly, we investigated whether hypoxia and/or high-glucose treatment had a detrimental effect on mitochondria in Neuro-2a cells. First, we examined the effect of hypoxia and/or high glucose on Δψm via JC-1 staining. Quantitative analysis by flow cytometry revealed an increase in the percentage of cells with reduced Δψm, as judged by the lower ratio of red to green fluorescence under hypoxia (Fig. 6a). However, Δψm did not change dramatically under high glucose conditions. Second, we evaluated alterations in mitochondrial structure during chronic hypoxia by TEM. As shown in Fig. 6b, mitochondria displayed features of swelling, dilation and disruption of cristae under hypoxia, illustrating an obvious toxic effect of chronic hypoxia on mitochondria. Moreover, the toxic effect of hyperglycaemia might be negligible under our experimental conditions.

### Insufficient BNIP3-mediated mitophagy during chronic hypoxia

**Figure 7:**
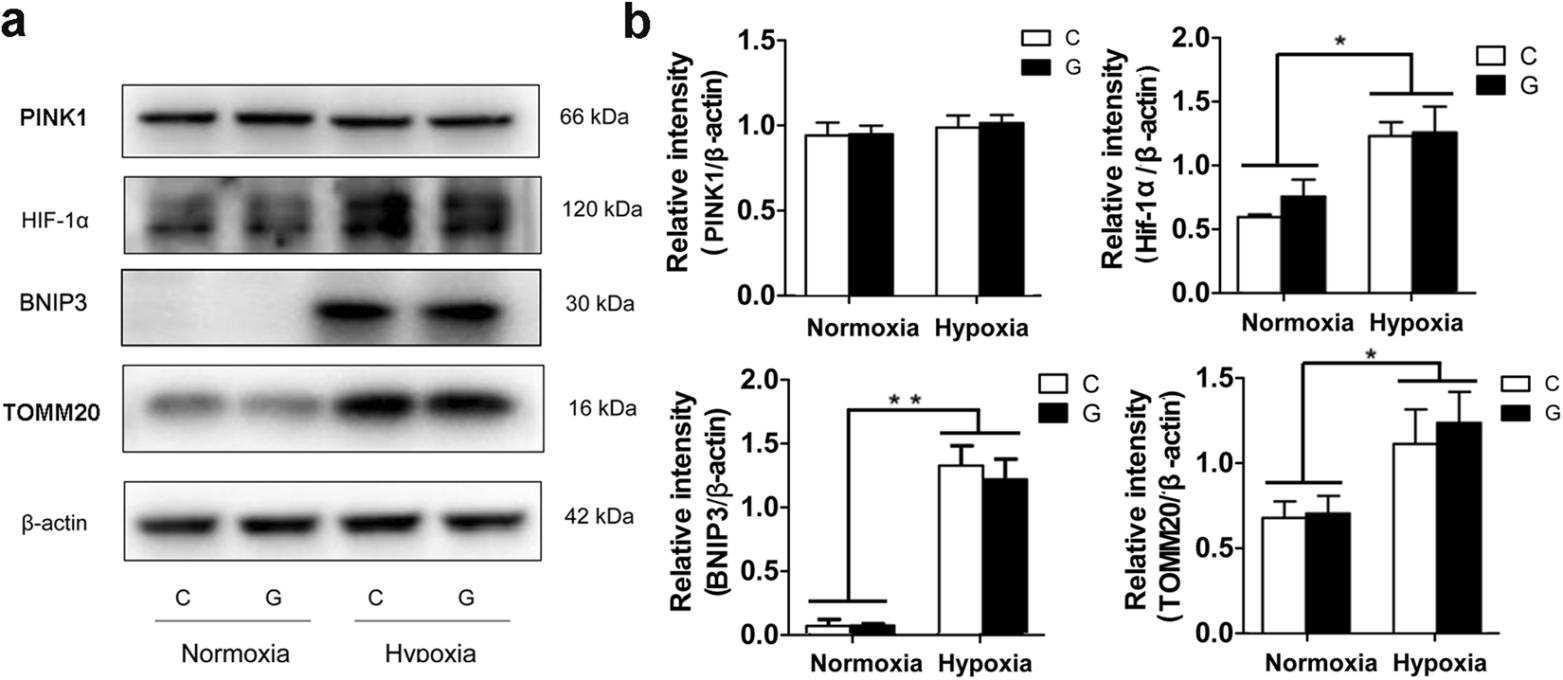
The HIF-1α-BNIP3 pathway was activated during hypoxia. (**a**) Analysis of mitophagy-related protein expression in Neuro-2a cells by immunoblotting. Neuro-2a cells were exposed to hypoxia and/or high glucose for 48 h. Total cellular extracts were analysed by immunoblotting with antibodies against HIF-1α, BNIP3, PINK1 and TOMM20. (**b**) The ImageJ densitometric analysis is shown. “C” represented control conditions (25 mM glucose), “G” represented high glucose conditions (75 mM glucose). The results are expressed as the mean ± SEM from three independent experiments. Two-way ANOVA, **p < 0.01, *p < 0.05, Hypoxia vs Normoxia. Full-length blots are presented in Supplementary Fig. S2.

Once the mitochondrial outer membrane becomes permeabilized and Δψm is reduced, injured mitochondria release apoptogenic factors, especially cytochrome c, with eventual caspase activation to invoke apoptosis. Thus, removing damaged mitochondria to maintain an appropriate mitochondrial quality is essential for cells. Mitophagy, which refers to the selective clearance of injured mitochondria by autophagy machinery, is an exact mitochondrial control process. To determine whether mitophagy is important to Neuro-2a cells under hypoxia and/or high glucose, we accessed two of the described mitophagy pathways: the PINK1-Parkin and HIF-1α-BNIP3 pathways. The former has been considered as a response to the loss of Δψm, and the latter is thought to be associated with the removal of mitochondria under hypoxia. Notably, we found the HIF-1α-BNIP3 pathway to be effectively induced in response to hypoxia, as demonstrated by a clear increase in the levels of HIF-1α and BNIP3 proteins (Fig. 7). Surprisingly, immunoblot analysis showed that the protein level of PINK1 was not altered under hypoxia, suggesting that the PINK1-Parkin pathway might not be involved in Neuro-2a cells under our experimental conditions. In contrast to the observed activation of the HIF-1α-BNIP3 pathway, upregulated expression of translocase of outer mitochondrial membrane 20 (TOMM20), a mitochondrial membrane protein, was also found under hypoxia, indicating that BNIP3-mediated mitophagy might not be sufficient to eliminate mitochondria during hypoxia.

**Figure 8:**
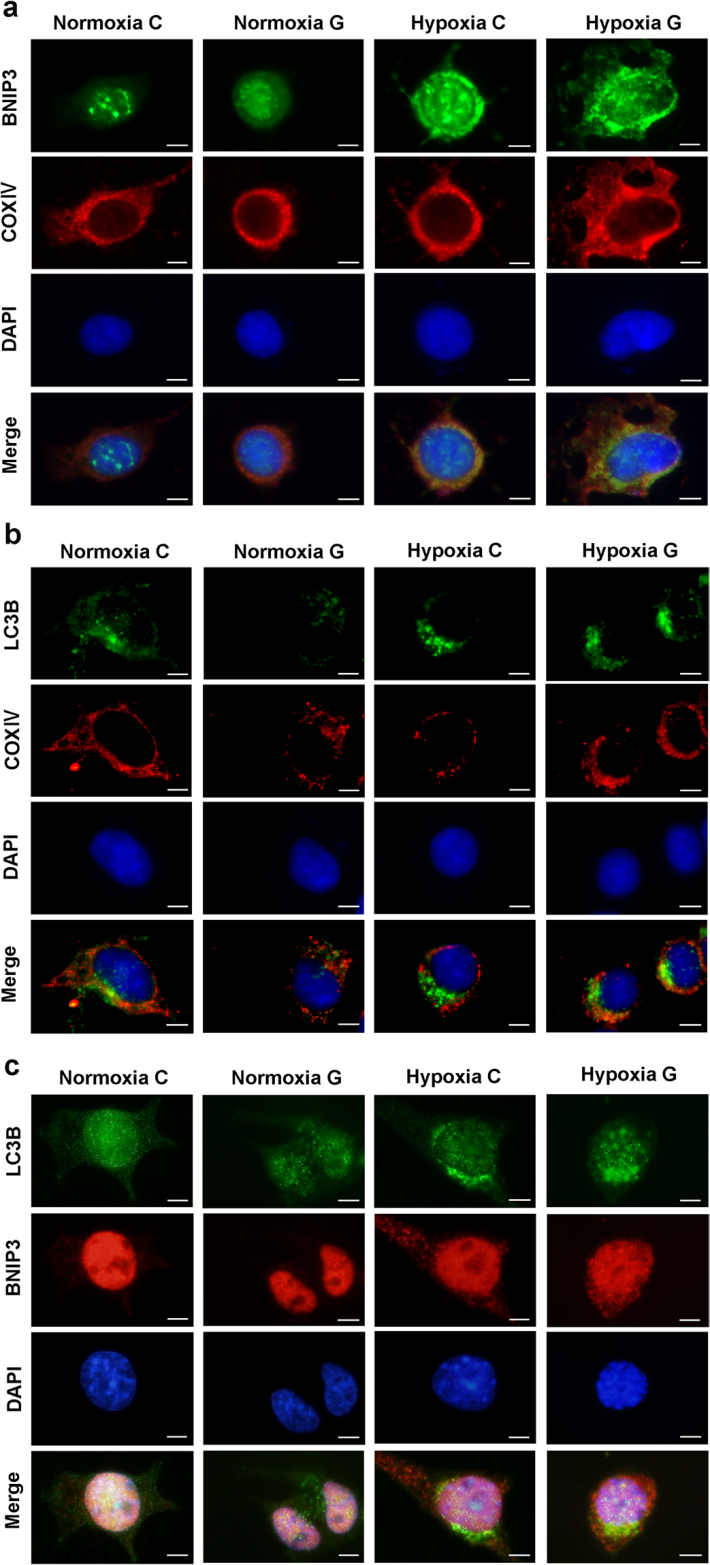
BNIP3-mediated mitophagy was insufficient during hypoxia. Neuro-2a cells were incubated for 48 h in each experiment, then fixed and immunostained with the LC3B, BNIP3, and COXIV antibodies. Cells were counterstained with DAPI to visualize nuclei. (**a**) Immunodetection of endogenous BNIP3 (green) colocalized with the mitochondrial marker COXIV (red), where it appeared as yellow. Scale bar: 50 μm. (**b**) Less colocalization of LC3B (green) and COXIV (red) was detected by fluorescence microscope. Scale bar: 50 μm. (**c**) Less colocalization of LC3B (green) and BNIP3 (red) was detected by fluorescence microscope. Scale bar: 50 μm.

To further clarify this discrepancy, we employed immunocytochemistry to detect the distribution and localization of mitophagy-related proteins. As shown in Fig. 8a, under hypoxia, green fluorescence-labelled BNIP3 appeared as distinct dot-like structures distributed in the cytoplasm and in the perinuclear region, which was consistent with the marked increase in BNIP3 protein during hypoxia shown on the immunoblot. In addition, colocalization of the green and red fluorescence(labelling the mitochondrial marker cytochrome c oxidase COXIV) signals, which appears yellow in the merged image, indicated a strong localization of BNIP3 in mitochondria. In contrast, the free green fluorescence signal(an indicative of accumulated LC3B protein under hypoxia) did not colocalize with the red fluorescence signal (labelling the mitochondrial marker, COXIV) in the merged image in Fig. 8b, suggesting the dysfunction of the mitophagy process. To test this hypothesis, we also examined the colocalization of green fluorescence-labelled LC3B and red fluorescence-labelled BNIP3 under hypoxic conditions. As expected, these proteins were not well-colocalized, as demonstrated by the fewer number of yellow dots in the cytoplasm (Fig. 8c).

**Figure 9:**
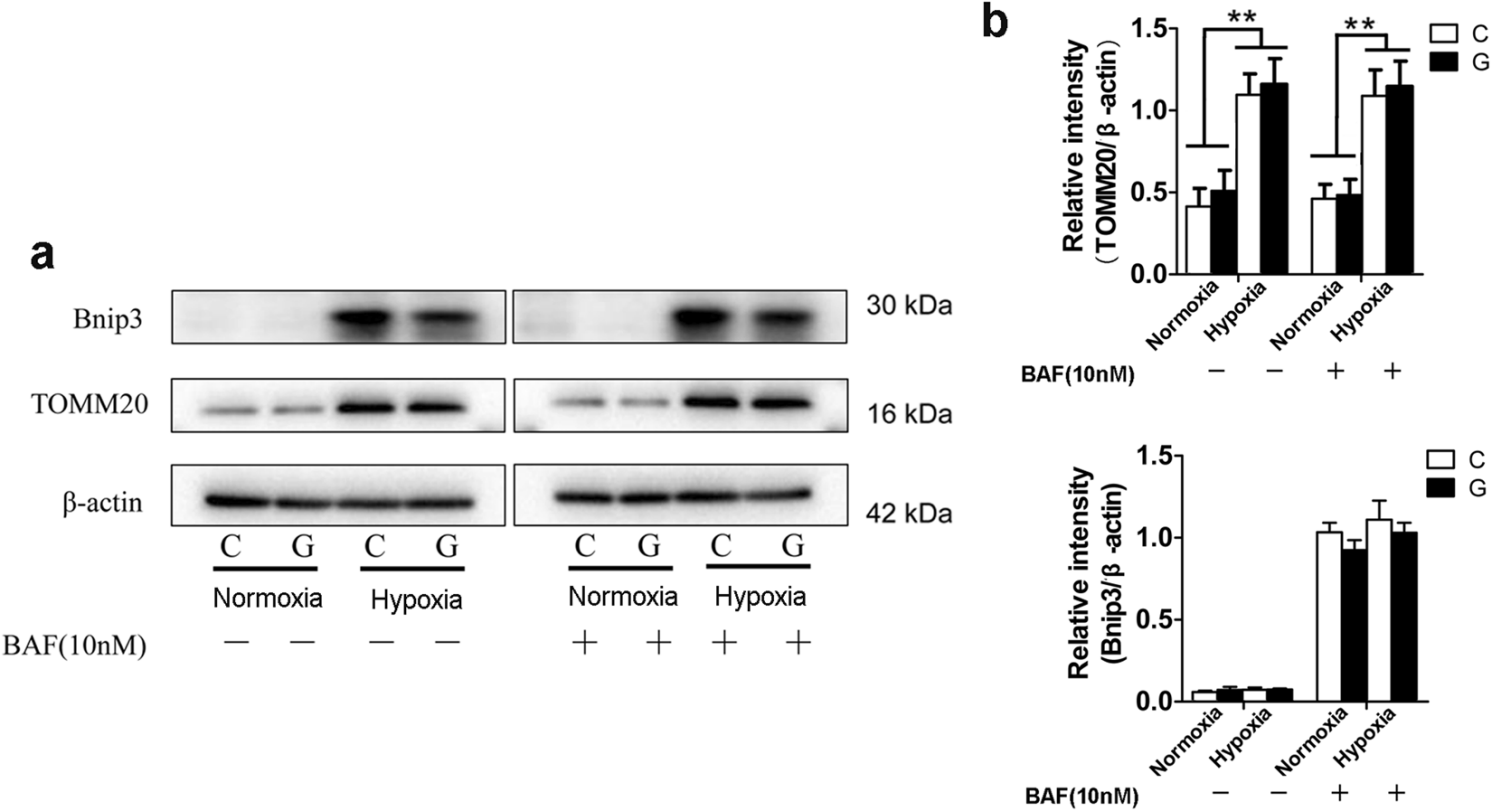
The accumulation of damaged mitochondria under hypoxia was independent of autophagy impairment. (**a**) Neuro-2a cells were submitted to hypoxia and/or high glucose for up to 42 h and then subjected to 10 nM BAF for an additional 6 h. Immunoblots were performed to determine the levels of BNIP3 and TOMM20. (**b**) The ImageJ densitometric analysis from immunoblots is shown. “C” represented control conditions (25 mM glucose), “G” represented high glucose conditions (75 mM glucose). The results are expressed as the mean ± SEM from three independent experiments. Two-way ANOVA, **p < 0.01, *p < 0.05, Hypoxia vs Normoxia. Full-length blots are presented in Supplementary Fig. S2.

To determine whether the impaired autophagy degradation under hypoxia was associated with the accumulation of damaged mitochondria, Neuro-2a cells were exposed to hypoxia and/or high glucose for up to 42 h and then treated with10 nM BAF for an additional 6 h. Notably, western blot analysis clearly showed that BAF treatment did not change the protein levels of BNIP3 and TOMM20 (Fig. 9). Collectively, these data suggest that a response of BNIP3-mediated mitophagy might not be sufficient in Neuro-2a cells exposed to hypoxia, which may in turn contribute to accumulation of injured mitochondria in cells.

## Discussion

In our study, we investigated whether high glucose exacerbates the pathologies of CCH and whether the mechanisms of autophagy and mitophagy are involved. Neuro-2a cells were treated with chronic hypoxia and/or high glucose *in vitro* to mimic the *in vivo* metabolic states. An MTS assay and immunoblot analysis of cleaved caspase-3 were employed to assess Neuro-2a cell viability. Immunoblot analysis of autophagy- and mitophagy-related proteins and observations of AVs and mitochondrial structure via fluorescence microscopy and TEM were used to detect autophagy and mitophagy changes. Taken together, our findings suggest that high glucose does not synergistically augment the cytotoxic effect of chronic hypoxia on Neuro-2a cells and that impaired autophagy degradation and inefficient BNIP3-mediated mitophagy may be two possible mechanisms underlying neuronal death during CCH.

Elderly DM patients commonly experience CCH. Several studies have observed abnormal low blood flow in cerebral grey matter tissue in type 2 DM patients, which can be regarded as evidence of cerebral hypoperfusion in DM^21,22^. Moreover, chronic hyperglycaemia has been shown to damage brain cells via a decrease in regional blood flow, an increase in membrane permeability and induction of ROS production^23^. One study reported that a mild increase in glucose (45 mM) induced cleavage of caspase-3 and apoptosis in cultured dorsal root ganglion neurons^24^. Another study demonstrated that culturing cells with high concentration of D-glucose (50–150 mM) for 7 days, but not 3 days, induced cell death in a dose-related manner^25^. Hyperglycaemia has been shown to increase neuronal glucose levels (up to fourfold) in DM patients^6^; therefore, we added 75 mM glucose to the medium (yielding a total of 100 mM glucose) for the high-glucose conditions in our study. Nevertheless, based on our results, treatment with high glucose for 48 h was not able to cause dramatic Neuro-2a cell death under our conditions, nor did it exacerbate the cytotoxicity caused by chronic hypoxia. This discrepancy can be explained by the following possibilities. One is that the Neuro-2a cells used in our study may be more resistant to the metabolic stress of high glucose than primary cultured neurons, and/or exposure to elevated glucose for 48 h may not be sufficient to significantly induce cell dysfunction and eventually cell death.

Several studies have demonstrated a neuroprotective role for autophagy in acute hypoxic/ischaemic brain injury^26,27^. Hypoxia caused by compromised blood supply results in phosphorylation and activation of AMPK, which can also suppress phosphorylation of mTOR, can directly or indirectly promote autophagy induction. Such an upregulation of autophagy supports neuronal survival via elimination of unnecessary intracellular materials^28^. Our study also found that chronic hypoxia induced an increase in AMPK phosphorylation and a decrease in mTOR phosphorylation, which coincided with an elevated level of the autophagy marker LC3-II, indicating autophagy initiation. Unexpectedly, another autophagy marker, P62, which is negatively associated with autophagy, was concomitantly upregulated, suggesting that the AVs formed during chronic hypoxia may be attributed to both induction and blockage of autophagy^29^. To clarify this, we treated Neuro-2a cells with an inhibitor of autophagy degradation, BAF^20^. Notably, BAF triggered a striking increase in LC3-II and P62 protein levels under normoxia, though no effect was found under hypoxia, suggesting that chronic hypoxia blocked autophagy prior to BAF treatment. In other words, chronic hypoxia had the same impact in inhibiting autophagic flux as that of BAF. All of these data suggest that chronic hypoxia may have a detrimental effect, similar to that of BAF, at least partially through impairment of autophagy in Neuro-2a cells. Pathological accumulation of autophagosomes in the brain has been demonstrated in AD, which is characterized by progressive cognitive decline. Many studies have reported that abnormal AV transport or acidificationis is responsible for the inefficient clearance of autophagosomes by lysosomes, which aggravates the burden of toxic proteins such as Aβ and exacerbates memory impairment^10^. Moreover, a recent study in an AD mouse model demonstrated that chronic hypoxia-induced autophagy may exacerbate neuropathological progression via the overwhelming burden of AVs^30^. Notably, the results of our study suggest that chronic hypoxia, a typical feature of CCH noted in AD and VaD^31^, may efficiently block autophagic degradation and thus amplify the development of pathologies and cognitive decline. Our study also showed that high glucose limited the activity of AMPK in both the presence and absence of hypoxia, potentially explaining the lower expression of LC3-II in cells treated with hypoxia and high glucose than in those treated with hypoxia alone. Given that AMPK is a nutrient sensor, it is not surprising that high glucose, a condition of nutrient excess, would inhibit the phosphorylation of AMPK and thus restrain the level of autophagy^32,33^.

Dysregulation of mitochondrial quality control has been linked to several neurodegenerative diseases. However, few studies have focused on the role of mitophagy in cognitive impairment diseases such as AD and VaD^34,35^. In the present study, we treated Neuro-2a cells with hypoxia and/or high glucose and assessed the role of mitophagy during the process. It has recently been demonstrated that mitophagy shares many steps with general autophagy, except for the specific recognition and selective engulfment of target mitochondria. One of the most studied mechanisms of mitophagy is the PINK1-Parkin pathway, in which the two main proteins PINK1 and Parkin are involved in the response to eliminate depolarized mitochondria. In our study, we observed a loss in Δψm and destruction of the mitochondrial structure in Neuro-2a cells during hypoxia. Unexpectedly, the protein level of PINK1 was not significantly altered, indicating that the PINK1-Parkin pathway might not be activated in Neuro-2a cells under hypoxia. An explanation for this observation is that a mild reduction in Δψm may not be sufficient to initiate the PINK1-Parkin pathway in these cells^20^. In addition, high glucose did not cause an obvious detrimental effect on mitochondria, suggesting that its neurotoxic role might be negligible under our experimental conditions. BNIP3-mediated mitophagy has been well studied under hypoxia, with the protein being upregulated by hypoxia-inducible factor-1 (HIF-1) in response to low oxygen levels^36^. In keeping with these observations, we found increased protein levels of HIF-1α and BNIP3 under hypoxia. Mitophagy can also be assessed by visualization of the colocalization of mitochondrial markers with autophagosome markers through microscopy analysis. Consistent with the increased level of BNIP3 protein shown on immunoblots, we found an enhanced BNIP3 localization to mitochondria by microscopy. However, LC3B was neither well colocalized with mitochondria nor with BNIP3, indicating a possibly inefficient mitophagy process caused by decoupling of LC3B and BNIP3. Additionally, we observed an increase in the protein level of mitochondrial marker TOMM20 under hypoxia. To further clarify that this result was not due to the blockage of autophagy, Neuro-2a cells were treated with BAF, and we found that BAF did not change the protein levels of BNIP3 or TOMM20. In summary, inefficient BNIP3-mediated mitophagy might be a reason for the accumulation of injured mitochondria in Neuro-2a cells during chronic hypoxia. The interaction of BNIP3 with LC3-IIthrough its LIR region is a rate-limiting step during BNIP-mediated mitophagy and can be regulated under certain conditions. One study reported that phosphorylation of serine residues 17 and 24, which flank the BNIP3 LIR domain, promotes interaction between BNIP3 and LC3, increasing the maturation and autophagic degradation of mitochondria^37^. Another study reported that disruption of the BNIP3 dimerized structure preserves its mitochondrial localization but fails to induce mitophagy^38^.

In conclusion, we show that high glucose does not synergistically augment the cytotoxic effect of chronic hypoxia on Neuro-2a cells. Furthermore, impaired autophagy degradation and inefficient BNIP3-mediated mitophagy may be two possible mechanisms underlying neuronal death during chronic hypoxia, suggesting a potential mechanism for the pathogenesis of CCH-related dementia.

## Materials and Methods

### Cell culture

Mouse neuroblastoma Neuro-2a cells (a gift from Prof Wedong Le, Shanghai University of Medicine and Health Sciences, passage number 5 since received)were cultured in Dulbecco’s modified Eagle’s medium (HyClone, USA) containing 10% foetal bovine serum (Gemini Bio-Products, Canada) and 1% penicillin streptomycin (HyClone, USA), under a humidified atmosphere at 37 °C in an incubator containing 5% CO_2_.

### Hypoxia and high-glucose treatment

For hypoxia treatment, Neuro-2a cells were incubated in an O_2_/N_2_/CO_2_ incubator (MCO-5M, Sanyo, Japan) and exposed to 1% O_2_ for the indicated time periods. The basal medium containing 25 mM glucose (control conditions, “C”) met the high metabolic requirements of neuronal cells. For high-glucose analyses(“G”), the medium was replaced by fresh medium with an additional 75 mM of glucose (Sigma,USA) to yield a total of 100 mM glucose. The osmolarities of all of media were adjusted to 100 mM by adding different amounts of mannitol (Sigma, USA).

### Antibodies and reagents

The antibodies used in this study were purchased from the following vendors: CST (USA)-LC3B, cleaved caspase-3, AMPK, P-AMPK, mTOR, P-mTOR; Abcam (UK)-P62, BNIP3; Sigma (USA)-LC3B-II; Novus (USA)-PINK1, HIF-1α; Santa Cruz(USA)-BNIP3; Proteintech (China)-TOMM20, COX IV, β-actin, anti-mouse IgG, anti-rabbit IgG, HRP-linked antibody; Invitrogen (USA):-Alexa Fluor 488 donkey anti-Rabbit IgG(H + L), Alexa Fluor 594 donkey anti-Mouse IgG(H + L). Bafilomycin A1 (Sigma, USA) was dissolved in dimethyl sulfoxide (DMSO; Sigma, USA) and added to media to the final concentrations described.

### Assessment of cell viability

Cell viability was measured using the CellTiter 96® AQueous Non-Radioactive Cell Proliferation Assay (MTS) (Promega, USA) according to the manufacturer’s instructions. Briefly, Neuro-2a cells were seeded in white-walled clear-bottom 96-well plates with a cell density of 1 × 10^5^/ml. After culturing overnight, the cells were treated with hypoxia and/or high glucose for 48 h. Upon addition of the MTS solution, the plate was incubated at 37 °C for 2 h, and absorbance was measured at 490 nm using a plate reader (TECAN, Männedorf, Switzerland). Cells were treated in triplicate, and data were normalized to the control; the results are reported as the percentage of viable cells.

### Western blotting

Neuro-2a cells were lysed in ice-cold RIPA Lysis Buffer. The protein concentration of each sample was determined by the bicinchoninic acid (BCA) protein assay (Pierce Biotechnology, Rockford, IL, USA). The samples were denatured by adding 5 × SDS-PAGE Sample Loading Buffer (Beyotime institute of biotechnology, China) and heating for 10 min at 100 °C. Equal amounts of protein were separated by10%–15% sodium dodecyl sulfate-polyacrylamide gel electrophoresis (SDS-PAGE) and then transferred electrophoretically to polyvinylidene fluoride(PVDF) membranes (Millipore, Billerica, MA, USA). After blocking with 5% non-fat milk in Tris-buffered saline (50 mM TRIS-HCl, pH 7.5, 150 mM NaCl) containing 0.2% Tween 20, the membranes were probed with antibodies against the following proteins: LC3B (Sigma, L7543), SQSTM1/P62 (Abcam, ab56416), cleaved caspase-3 (CST, 9661), HIF-1α (Novus, NB100-105), PINK1 (Novus, BC100-494), BNIP3 (Abcam, ab109362), and β-actin (Proteintech, 60008-1-Ig). Following incubation with secondary anti-mouse (Proteintech, SA00001-1) or anti-rabbit (Proteintech, SA00001-2)antibodies, protein bands were visualized using enhanced chemiluminescence(ECL) blotting detection reagents (Millipore, WBKLS0500).

### TEM

Cell samples were processed by the Electron Microscopy Core at Fudan University^39^. Cell pellets were fixed with 2.5% glutaraldehyde in 0.1 M phosphate buffer and then washed with 0.1 M sodium cacodylate buffer and post fixed with 1% osmium tetroxide. The pellets were dehydrated in a graded ethanol series, infiltrated, and embedded in Spurr’s resin. Samples were polymerized for 48 h at 60 °C, cut into 60-nm-thick sections using an LKB-I microtome, positioned on 200-mesh grids, and stained with uranyl acetate and lead citrate. TEM was performed using a PHILIPS CM120 TEM at an accelerating voltage of 120 kV. Images were acquired with a connected Gatan type Ultra Scan 4000SP CCD camera.

### Fluorescence microscopy

Cells were washed twice with cold phosphate-buffered saline (PBS), fixed with cold acetone for 10 min at −20 °C, rinsed twice more in cold PBS, and then permeabilized with 0.25% Triton X-100 in PBS for 10 min at room temperature. After blocking for 30 min with 5% bovine serum albumin (BSA) in PBS, the cells were incubated with a primary antibody against LC3B (CST, 2775), BNIP3 (Abcam, ab109362), BNIP3(Santa Cruz, sc-56167), or COXIV (Proteintech, 60251-1-Ig) for 2 h at room temperature. The cells were rinsed three times with PBS + Tween 20 (PBST) and incubated for 1 h at room temperature in the dark with an Alexa Fluor 594-conjugated goat anti-mouse IgG secondary antibody (Invitrogen, A-11032) or Alexa flour 488-conjugated goat anti-rabbit IgG secondary antibody (Invitrogen, A-11034).DAPI (4′,6-diamidino-2-phenylindole)was used to stain nuclei. The preparations were visualized using a Zeiss confocal microscope (LSM 510, Germany). ForJC-1 staining (Beyotime institute of biotechnology, China), live cell imaging using 2.5 μM JC1 was performed on stained cells in Dulbecco’s modified Eagle’s medium with 10% FBS.

### Flow cytometry

Neuro-2a cells were treated with hypoxia and/or high glucose for 48 h, and the JC-1dye was used to measure Δψm (Beyotime institute of biotechnology, China). At low concentrations, the dye exists as a monomer with green fluorescence (E_m_ 530 nm) and accumulates in mitochondria as aggregates with orange-red fluorescence (E_m_ 590 nm). When mitochondria are damaged, depolarization of the mitochondrial membrane potential can be monitored by the shift in fluorescence from red to green. Briefly, cells were treated as described above, harvested and incubated with JC1 working solution for 20 min at 37 °C according to the manufacturer’s instructions. The cells were then washed, resuspended in fresh medium and analysed using a FACS Caliber flow cytometer (BD Biosciences, San Jose, CA). Data were evaluated using FlowJo software (Tree Star Inc, Ashland, OR), and the results are represented as a dot plot of green versus red fluorescence. Δψm depolarization was determined by an increase in red fluorescence (aggregates) accompanied by a loss in green fluorescence (monomers).

### Statistical analysis

Data are expressed as the mean ± SEM from three independent experiments. Statistical analyses were performed using the two-way analysis of variance (ANOVA). Values of *P < 0.05 were considered significant.

### Data availability statement

The datasets generated during and/or analysed during the current study are available from the corresponding author on reasonable request.

## Electronic supplementary material

supplementary information
