# Supplementary material for: Involvement of impaired autophagy and mitophagy in Neuro-2a cell damage under hypoxic and/or high-glucose conditions
Source: Sci Rep. 2018 Feb 19;8:3301. doi: 10.1038/s41598-018-20162-1 (PMC5818622; doi:10.1038/s41598-018-20162-1)
Supplement: Supplementary file 1 — supplementary information [file 41598_2018_20162_MOESM1_ESM.pdf]

Involvement of impaired autophagy and mitophagy in Neuro-2a cell damage under hypoxic and/or high-glucose conditions

Yufei Song, MD[1, #]; Yu Du , MD[1, #]; Wenying Zou, MD[1]; Yan Luo,MD[1]; Xiaojie Zhang, MD, PhD[1,*]; Jianliang Fu, MD, PhD[1,*].

[1]Department of Neurology, Shanghai Jiao Tong University Affiliated Sixth People's Hospital, 600 Yishan Road, Shanghai 200233, China

# Yufei Song and Yu Du contributed equally to this manuscript

*Jianliang Fu and Xiaojie Zhang were the co-correspondence authors

Address correspondence to:

Jianliang Fu

Department of Neurology,

Shanghai Jiao Tong University Affiliated Sixth People's Hospital,

600 Yishan Road,

Shanghai 200233, China

Telephone: +86 18930177585 FAX:021 - 24058354

E-mail: fujianliang@163.com

Supported by the Project of National Natural Science Foundation of China (No. 81672243) and Shanghai Municipal Planning Commission of Science and Research Fund (No.20164Y0074).

Word count: 4002 words

Number of figures: 9

Figure S1

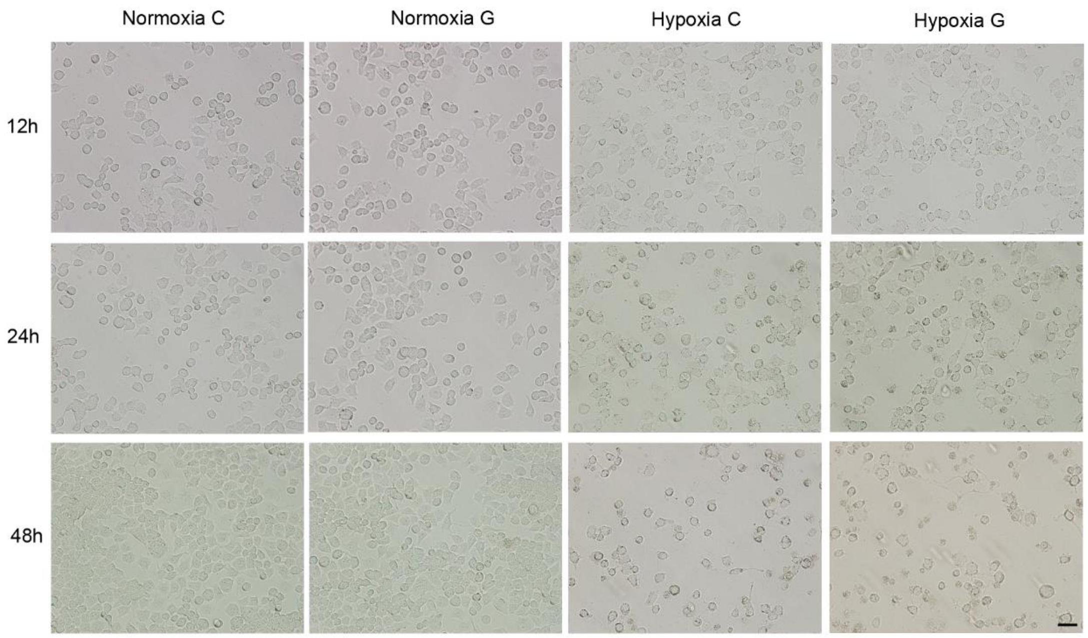

Figure S1. Neuro-2a cells were subjected to hypoxia and/or high glucose for up to 48 h, and the morphologies of Neuro-2a cells at different time points are shown in the bright field images. Scale bar, 50 μm.

Figure S2. Full scans of original Western blots for data in Figure 1, 2, 4, 5, 7, 9. Panels crossed in red corresponding to the figures in the paper are indicated.

Figure 1b

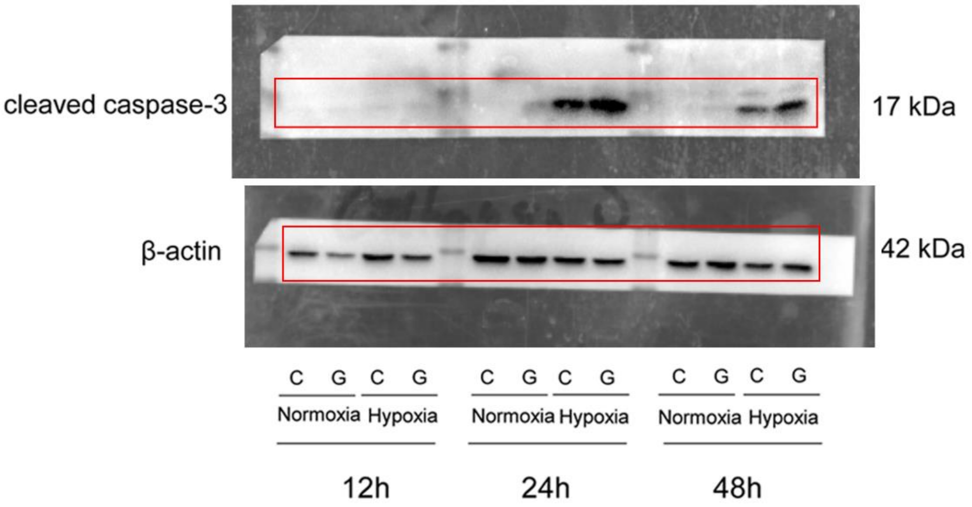

Figure 2a

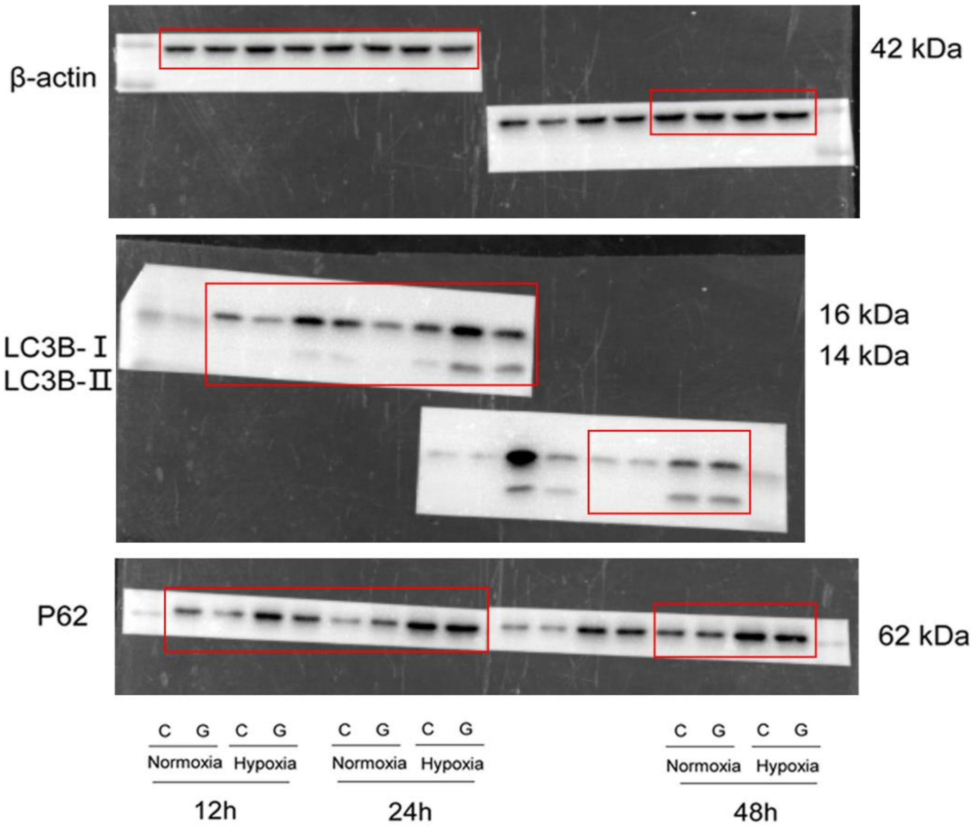

Figure S2 continued

Figure 4a

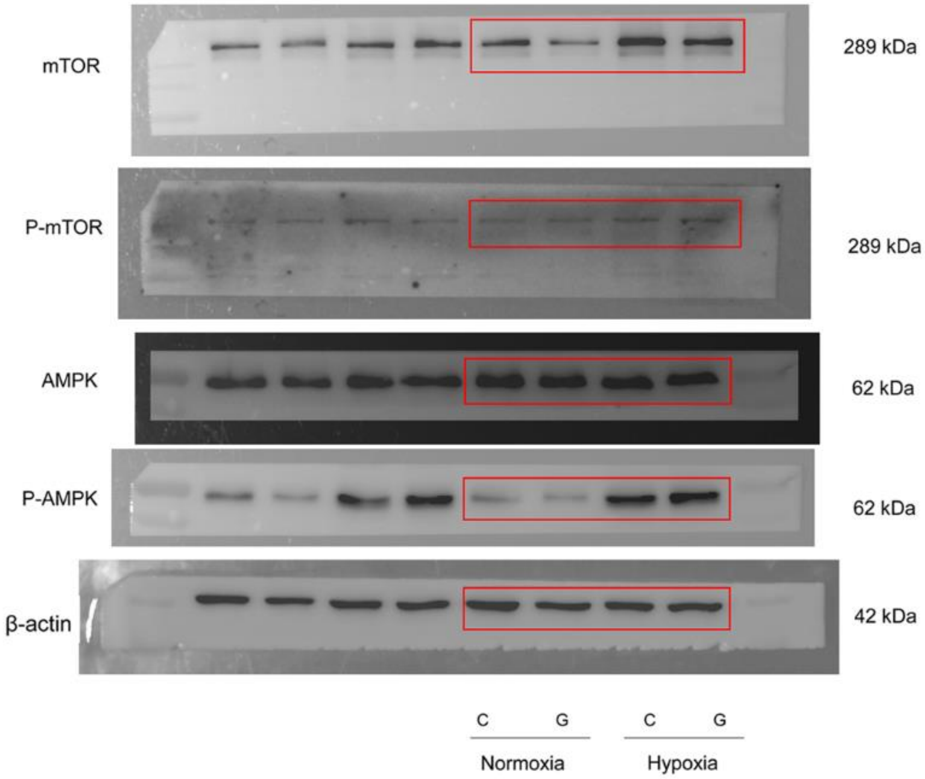

Figure 4c

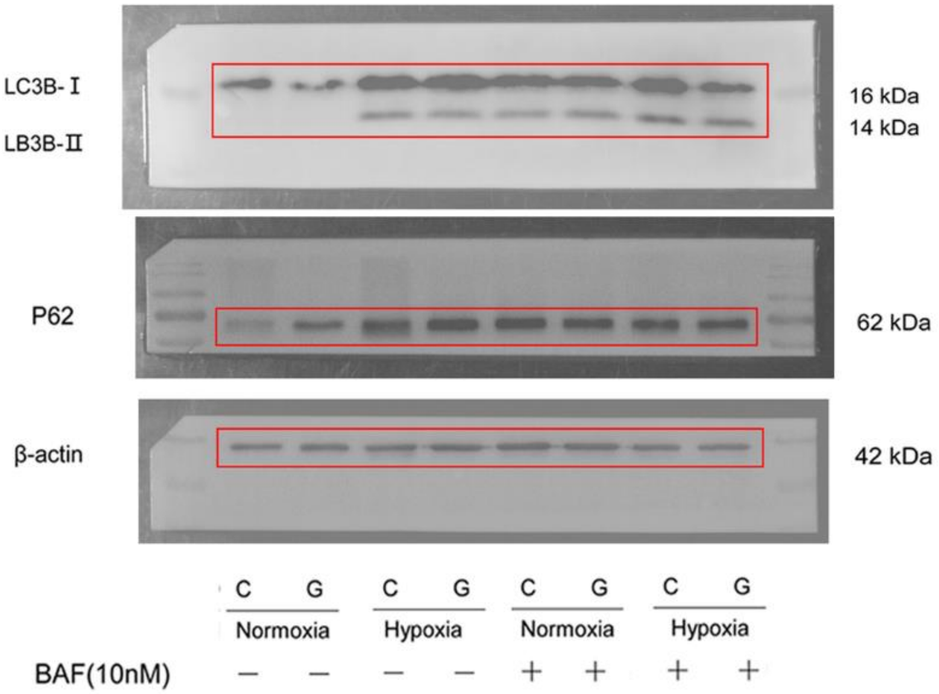

Figure S2 continued

Figure 5b

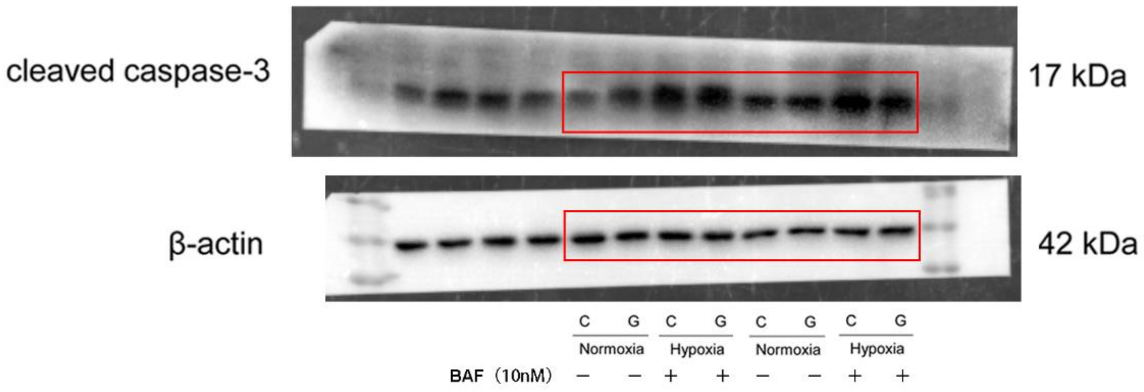

Figure 7a

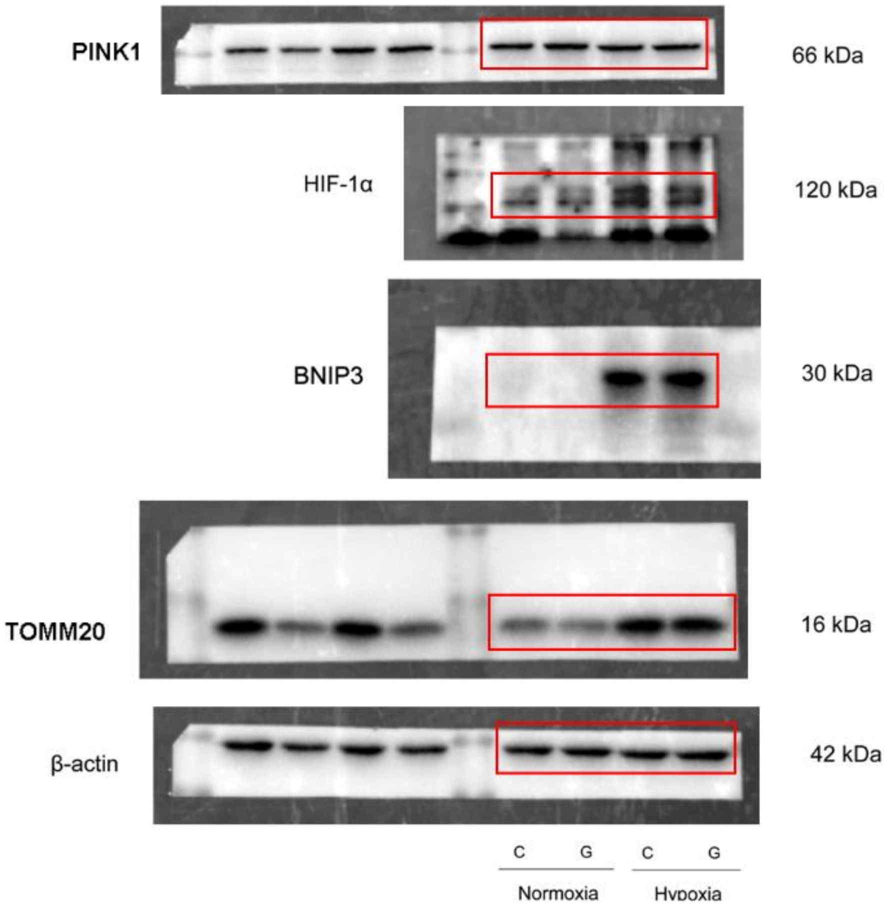

Figure S2 continued

Figure 9a

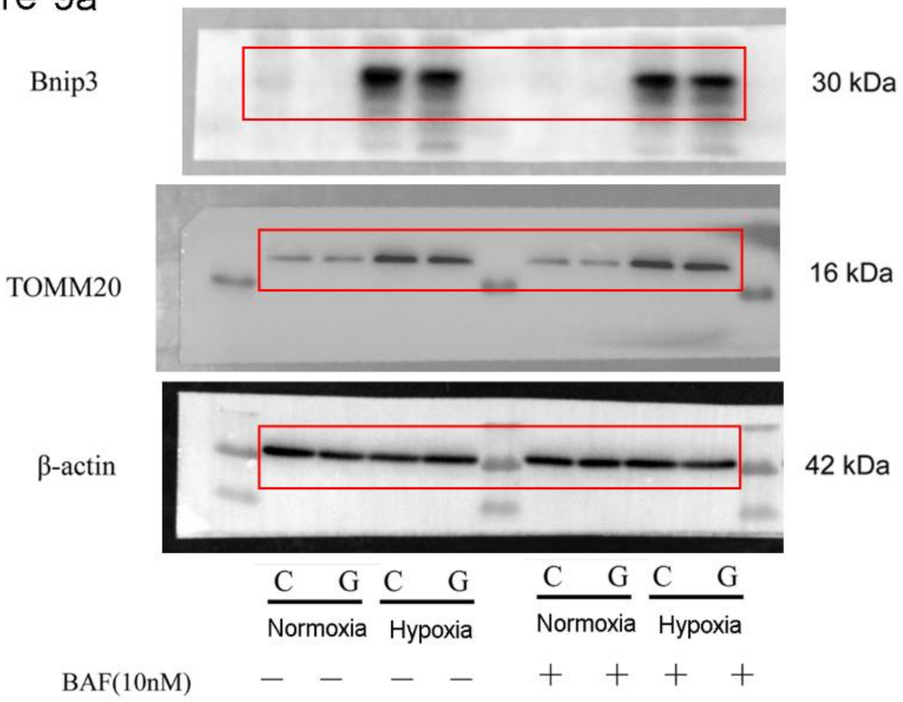
Bnip3
30 kDa
TOMM20
16 kDa
β-actin
42 kDa
C G C G C G C G
Normoxia Hypoxia Normoxia Hypoxia
BAF(10nM) – – – – + + + +
